# Implementation of tuberculosis prevention for exposed children, Burkina Faso

**DOI:** 10.2471/BLT.17.201343

**Published:** 2018-04-20

**Authors:** Giorgia Sulis, Adjima Combary, Haileyesus Getahun, Saidou Gnanou, Pier Francesco Giorgetti, Arnaud Konseimbo, Susanna Capone, Yohhei Hamada, Annabel Baddeley, Alberto Matteelli

**Affiliations:** a Department of Infectious and Tropical Diseases, University of Brescia, Piazzale Spedali Civili 1, 25123 Brescia, Italy.; b National Tuberculosis Programme, Ouagadougou, Burkina Faso.; c Global Tuberculosis Programme, World Health Organization, Geneva, Switzerland.; d L’Initiative Privée et Communautaire pour la Santé et la Riposte au VIH/SIDA, Ouagadougou, Burkina Faso.

## Abstract

**Objective:**

To develop and test a simple system for recording and reporting the diagnosis and treatment of latent tuberculosis infection and to compare the effects of passive and active tracing of child contacts on indicators of such infection.

**Methods:**

We revised Burkina Faso’s latent tuberculosis infection register and quarterly tuberculosis reporting form. Subsequently, coverage of the routine screening of contacts, who were younger than five years, for active tuberculosis and the corresponding percentages of such contacts who, if eligible, initiated preventive therapy were measured, nationwide, between 1 April 2016 and 31 March 2017. In 2016, we evaluated indicators of latent tuberculosis infection in the Hauts-Bassins region before and after community health workers had begun the active tracing of contacts who were younger than five years.

**Findings:**

In Burkina Faso, during our study period, 3717 cases of pulmonary tuberculosis and 1166 corresponding contacts who were younger than five years were reported as the result of routine screening and passive contact tracing. The overall contact:index ratio was 0.31 and corresponding screening coverage was 82.0% (956/1166) and proportion of children starting on preventive treatment was 90.5% (852/941). Active tracing in Hauts-Bassins led to a substantially higher contact/index ratio (1.83) and screening coverage (99.3%; 145/146).

**Conclusion:**

The newly established recording and reporting system proved feasible and user-friendly and allowed measurement of global indicators of latent tuberculosis infection. Compared with active tracing, passive tracing led to much lower estimates of the numbers of child contacts.

## Résumé

**Objectif:**

Élaborer et tester un système simple pour enregistrer et déclarer le diagnostic et le traitement des cas d'infection tuberculeuse latente et comparer les effets de la recherche passive et active des contacts enfants sur les indicateurs de cette infection.

**Méthodes:**

Nous avons passé en revue le registre des infections tuberculeuses latentes et le formulaire de rapport trimestriel des cas de tuberculose du Burkina Faso. Ensuite, nous avons mesuré, dans tout le pays, entre le 1^er^ avril 2016 et le 31 mars 2017, le taux de couverture du dépistage systématique de la tuberculose active chez les contacts âgés de moins de cinq ans et les pourcentages correspondants de contacts qui, s'ils remplissaient les conditions requises, avaient commencé un traitement préventif. En 2016, nous avons évalué les indicateurs de l'infection tuberculeuse latente dans la région des Hauts-Bassins avant et après que les agents de santé communautaires ont commencé la recherche active des contacts âgés de moins de cinq ans.

**Résultats:**

Au Burkina Faso, au cours de la période étudiée, 3717 cas de tuberculose pulmonaire et 1166 contacts correspondants âgés de moins de cinq ans ont été déclarés dans le cadre du dépistage systématique et de la recherche passive des contacts. Le ratio total contacts/cas index était de 0,31 et le taux de couverture du dépistage correspondant s'élevait à 82,0% (956/1166), tandis que la proportion d'enfants ayant commencé un traitement préventif était de 90,5% (852/941). La recherche active dans les Hauts-Bassins a entraîné une hausse sensible du ratio total contacts/cas index (1,83) et du taux de couverture du dépistage (99,3%; 145/146).

**Conclusion:**

Le nouveau système d'enregistrement et de déclaration s'est avéré utilisable et convivial et a permis de mesurer les indicateurs globaux de l'infection tuberculeuse latente. En comparaison avec la recherche active, la recherche passive a généré des estimations beaucoup plus faibles du nombre de contacts enfants.

## Resumen

**Objetivo:**

Desarrollar y probar un sistema simple de registro y presentación de informes sobre el diagnóstico y el tratamiento de la infección tuberculosa latente y comparar los efectos del seguimiento pasivo y activo de los contactos infantiles con indicadores de dicha infección.

**Métodos:**

Se revisó el registro de infección tuberculosa latente de Burkina Faso y el formulario trimestral de información sobre la tuberculosis. Posteriormente, se midió la cobertura de los exámenes de detección rutinarios a los contactos, menores de cinco años, para la tuberculosis activa y los porcentajes correspondientes de contactos que, de ser aptos, iniciaron la terapia preventiva a nivel nacional, entre el 1 de abril de 2016 y el 31 de marzo de 2017. En 2016, se evaluaron los indicadores de infección tuberculosa latente en la región de Hauts-Bassins antes y después de que los trabajadores comunitarios de la salud empezaran el seguimiento activo de los contactos menores de cinco años.

**Resultados:**

En Burkina Faso, en nuestro periodo de estudio, se registraron 3717 casos de tuberculosis pulmonar y 1166 contactos correspondientes de menos de cinco años como resultado de un examen rutinario y un seguimiento del contacto pasivo. La tasa global de índice de contacto fue del 0,31 y la cobertura de detección correspondiente fue del 82,0% (956/1166), además, la proporción de niños que iniciaron el tratamiento preventivo fue del 90,5% (852/941). El seguimiento activo en Hauts-Bassins dio lugar a una relación de contacto/índice (1,83) y una cobertura de detección (99,3%; 145/146) sustancialmente más altas.

**Conclusión:**

El recién establecido sistema simple de registro y presentación de informes resultó factible y fácil de usar y permitió la medición de indicadores globales de la infección tuberculosa latente. En comparación con el seguimiento activo, el seguimiento pasivo dio lugar a estimaciones mucho más bajas de la cantidad de contactos secundarios.

## ملخص

**الغرض:**

وضع واختبار نظام بسيط للتسجيل والإبلاغ عن حالات تشخيص وعلاج العدوى الكامنة بداء السل، ومقارنة آثار التتبع السلبي والنشط لحالات تعرض الأطفال إلى المرض بمؤشرات تلك العدوى.

**الأسلوب:**

لقد ****راجعنا سجل الإصابة بداء السل الكامن في بوركينا فاصو، ونموذج البلاغات ربع السنوية للإصابة بداء السل. ****وبعد ذلك، تم قياس حجم تغطية الفحص الدوري لحالات الاتصال بالمرض بين الأطفال دون سن خمس سنوات للكشف عن حالات الإصابة النشطة بداء السل وما يقابلها من نسب الاتصال بالمرض بين الأطفال الذين شرعوا في تعاطي العلاج الوقائي إذا كانوا مؤهلين له، وذلك على مستوى البلاد في الفترة ما بين 1 أبريل/نيسان 2016 و31 مارس/آذار 2017. وفي عام 2016، قمنا بتقييم مؤشرات الإصابة بداء السل الكامن في منطقة أوت باسان قبل وبعد قيام الأخصائيين الصحيين المجتمعيين بالبدء في عمليات التتبع النشط لحالات الاتصال بالمرض بين الأطفال الذين تقل أعمارهم عن خمس سنوات.

**النتائج:**

في بوركينا فاصو، تم الإبلاغ عن 3717 حالة للإصابة بداء السل الرئوي و1166 حالة مقابلة من الاتصال بالمرض بين الأطفال الأقل من خمس سنوات – كنتيجة للفحص الدوري وعمليات التتبع السلبي – وذلك خلال فترة الدراسة. وبلغت النسبة الإجمالية لحالات التعرض للمرض إلى مؤشر الإصابة 0.31، أما النسبة المقابلة لشمول خدمات الفحص فبلغت نسبتها 82.0‏% (بواقع ‏956/1166)، وبلغت نسبة الأطفال الذين بدأوا العلاج الوقائي 90.5‏% (852/941). وقادت عمليات التتبع النشط في منطقة أوت باسان إلى ارتفاع ملحوظ في نسبة حالات التعرض للمرض إلى مؤشر الإصابة (1.83)، أما نسبة شمول خدمات الفحص فبلغت (99.3‏%؛ 145/146).

**الاستنتاج:**

أثبت النظام الذي تم تأسيسه مؤخرًا لتسجيل حالات الإصابة وتقديم البلاغات عنها جدواه وسهولة استخدامه، وسمح بقياس المؤشرات العالمية لحالات العدوى الكامنة بداء السل. وبالمقارنة مع عمليات التتبع النشط، فقد قاد التتبع السلبي إلى تقديرات أقل كثيرًا لأعداد الأطفال الذين تعرضوا إلى الاتصال بالمرض.

## 摘要

**目的:**

研发并测试一个简易系统，用于记录和报告潜在结核病感染的诊断与治疗，并对此类感染指标下针对儿童接触者分别进行被动追踪和主动追踪的效果进行比较。

**方法:**

我们修订了布基纳法索潜在结核病感染登记和季度结核病申报表。随后，在 2016 年 4 月 1 日至 2017 年 3 月 31 日之间，针对年龄不足 5 岁的活动性结核病患者进行常规性筛查并对此类符合条件的接触者开始实施预防性治疗，分别测量其在全国范围内的覆盖率和相应的百分比。2016 年，我们先评估了上盆地大区潜在结核病感染指标，之后，社区健康工作者开始积极追踪年龄不足 5 岁的儿童接触者。

**结果:**

在我们研究期间，经过常规性筛查和被动接触追踪，据报告称，布基纳法索有 3717 例结核病案例和 1166 位年龄不足 5 岁的相应接触者。总体接触指数比为 0.31，相应的筛查覆盖率为 82.0%（956/1166)，开始预防性治疗的儿童比例为 90.5%（852/941)。对上盆地大区的结核病积极追踪导致更高的接触指数比 （1.83） 和筛查覆盖率 （99.3%，145/146）。

**结论:**

新建立的记录报告系统被证明行之有效且用户友好，其可以评估潜伏结核病感染的全球指标。与积极追踪相比，消极追踪会造成对儿童接触者数量的低估。

## Резюме

**Цель:**

Разработать и протестировать простую систему регистрации и отчетности для диагностики и лечения латентной туберкулезной инфекции и сравнить влияние пассивного и активного отслеживания детей, находившихся в контакте с инфицированным человеком, на показатели такой инфекции.

**Методы:**

Авторы изучили реестр лиц, больных латентным туберкулезом, в Буркина-Фасо и ежеквартальную форму отчетности о количестве больных туберкулезом. После этого по всей стране в период с 1 апреля 2016 года по 31 марта 2017 года была проведена оценка охвата регулярным скрининговым обследованием детей младше пяти лет, находившихся в контакте с инфицированным человеком, на предмет выявления активного туберкулеза и соответствующей доли таких детей, для которых при необходимости была назначена профилактическая терапия. В 2016 году мы оценили показатели латентной туберкулезной инфекции в области Верхних Бассейнов до и после того, как работники здравоохранения начали активное отслеживание детей младше пяти лет, находившихся в контакте с инфицированным человеком.

**Результаты:**

В Буркина-Фасо в течение периода исследования авторов в результате регулярного скрининга и пассивного отслеживания контактов было зарегистрировано 3717 случаев туберкулеза легких и 1166 соответствующих детей младше пяти лет, находившихся в контакте с инфицированным человеком. Общее соотношение «контакт/показатель» составило 0,31, соответствующий охват скрининговым обследованием — 82,0% (956/1166), а доля детей, для которых было назначено профилактическое лечение, — 90,5% (852/941). Активное отслеживание в области Верхних Бассейнов привело к значительно более высокому соотношению «контакт/показатель» (1,83) и охвату скрининговым обследованием (99,3%, 145/146).

**Вывод:**

Недавно созданная система регистрации и отчетности оказалась выполнимой и удобной для пользователей и позволила измерить глобальные показатели латентной туберкулезной инфекции. При сравнении с активным отслеживанием пассивное отслеживание привело к значительно более низким показателям количества детей, находившихся в контакте с инфицированным человеком

## Introduction

The identification and treatment of individuals with latent *Mycobacterium tuberculosis* infection is an essential element of the World Health Organization’s (WHO’s) End TB Strategy.^1^ Individuals with such infection, who are thought to make up between a third and a quarter of the world’s population, act as reservoir hosts and may develop into active cases of tuberculosis when reactivation is triggered by any of several known risk factors.^2^^,^^3^ Up to one third of the household contacts of humans with untreated tuberculosis of the respiratory tract become infected with *M. tuberculosis* and the risk of infection is relatively high if the active index case has severe disease, exposure to the case is prolonged and exposure to ultraviolet light and ventilation are poor.^3^ Compared with older children, infants and very young children with latent infection are more likely to develop active tuberculosis, especially the severe and life-threatening forms of the disease.^3^ Recommendations therefore suggest that all children younger than five years who are household contacts of cases of pulmonary tuberculosis should be screened for active tuberculosis and, if found free of the active disease and otherwise eligible, given preventive treatment.^4^^,^^5^

The percentage of eligible child contacts who begin preventive treatment (hereafter called treatment coverage) is considered to be among the top 10 priority indicators for measuring the progress of the End TB Strategy at global and national levels.^6^ According to WHO, in 2016, an estimated 1.3 million children younger than five years were eligible for treatment for the prevention of tuberculosis.^7^ Although treatment coverage appears to have almost doubled between 2015 and 2016, it was estimated to be just 13% in 2016.^7^ Global figures on another indicator, the proportion of exposed children who are investigated to exclude active tuberculosis and, if eligible, start preventive therapy, are not yet available.

The screening and treatment of contacts younger than five years, as recommended by the WHO, probably occurs in most countries with a high burden of tuberculosis.^8^ In 2016– 2017, however, very few countries in WHO’s Africa Region reported having any kind of scheme for the monitoring and evaluation of their interventions against latent tuberculosis infection.^9^ In addition, very few such countries had any system for the routine reporting of four core indicators, i.e. the percentages of child contacts screened for active disease, eligibility for treatment for the prevention of tuberculosis, initiating such treatment and completing such treatment, that could give useful insights as to their interventions’ efficiency.^9^

Burkina Faso is a resource-constrained country in West Africa that has a national tuberculosis programme and health-information system that appear similar to those of many other countries in sub-Saharan Africa. In 2015, Burkina Faso was one of the African countries that had no system for the monitoring and evaluation of latent tuberculosis infection and related interventions.^9^ Although a national policy for the systematic screening and treatment of such infection among people living with human immunodeficiency virus (HIV) and children younger than five years was already in place, the country did not have an efficient relevant recording and reporting system and only noted the number of children who were started on treatment for the prevention of tuberculosis.^10^ The programmatic management of latent tuberculosis infection in children younger than five years was implemented through a passive contact-tracing system that probably missed many children who were eligible for preventive treatment.^10^

In 2016, in response to the shortcomings in Burkina Faso’s tuberculosis control initiatives, we modified two existing reporting forms and developed a monitoring and evaluation system for all of the country’s activities relating to latent tuberculosis infection. We analysed the data produced within the system over a 12-month period, i.e. from 1 April 2016 to 31 March 2017, and used the data to evaluate screening coverage and proportion of children starting on preventive treatment. At the time of our analyses, the corresponding data on treatment completion rates were not yet available. In a second study, we implemented a pilot project in one area of Burkina Faso to compare the active and passive tracing of contacts younger than five years and then documented the observed impacts of active tracing on some key indicators of latent tuberculosis infection.

## Methods

### Nationwide monitoring and evaluation

#### Recording and reporting system

**Fig. 1:**
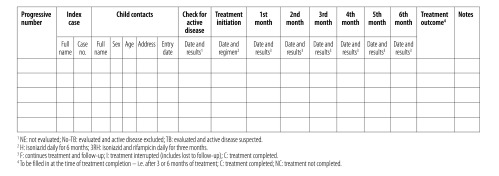
The national tuberculosis registration form – revised to record data on tuberculosis-case contacts younger than five years, Burkina Faso, 2016

Note: The text in the form used in Burkina Faso was in French.

In early 2016 Burkina Faso’s national tuberculosis programme, in collaboration with WHO and the University of Brescia, Italy, developed a simple register to document and report the numbers of children younger than five years who were household contacts of contagious tuberculosis cases and who were screened and, where appropriate, started and sometimes completed treatment for latent tuberculosis infection. This entailed revision of the existing national paper-based register of latent tuberculosis infection so that the register could be used to record data on each child who was younger than five years and had been declared as a close or household contact of a case of pulmonary tuberculosis that had been bacteriologically confirmed. All such children were registered and information on their eligibility for treatment, treatment initiation and treatment completion was included routinely (Fig. 1).

In March 2016, copies of the revised registration form were printed and distributed to each of Burkina Faso’s 88 centres for tuberculosis diagnosis and treatment. At the same time, as part of the programme for the prevention of childhood tuberculosis, a national workshop was organized for training at least one health-staff member from each centre on the management, recording and reporting of latent tuberculosis infection.

The new registration form came into effect on 1 April 2016 and, in this paper, we present the data from the first 12 months of the form’s use. As part of their routine activities, staff from the national tuberculosis programme regularly supervised the form’s use either during visits to the centres for tuberculosis diagnosis and treatment, or in telephone conversations with the centres’ staff.

The target population was defined as children younger than five years old who shared households with individuals who had smear-positive pulmonary tuberculosis. The identification and screening of such children for active tuberculosis, as well as the provision of six months of preventive treatment with isoniazid to each child found eligible for such treatment, were conducted in accordance with the recommendations of the national tuberculosis programme.^10^

Routinely, contact tracing was based on passive detection of eligible children, who were identified from information provided by the index cases, usually a parent or other close relative, and evaluated if brought to a centre for tuberculosis diagnosis and treatment, voluntarily, when invited by a health-care provider. Such children were clinically evaluated to exclude cough of at least two weeks’ duration, weight loss or failure to gain weight, fever, night sweats, fatigue and/or reduced playfulness. Wherever possible, if any of these symptoms were recorded, the child was checked for active tuberculosis via chest radiography and gastric lavage to collect samples that were checked by smear microscopy and/or rapid diagnostic testing (Xpert® MTB/RIF; Cepheid, Sunnyvale, United States of America). All child contacts without active disease were considered eligible for isoniazid preventive therapy.

#### Indicators

**Fig. 2:**
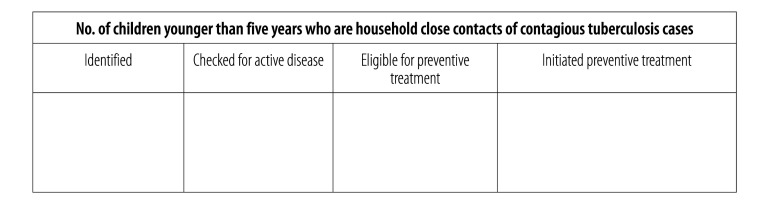
The record block added to the national quarterly reporting form for tuberculosis to record data on tuberculosis-case contacts younger than five years, Burkina Faso, 2016

Note: The text in the form used in Burkina Faso was in French.

In March 2016, the pre-existing quarterly tuberculosis reporting form was modified to include information on the numbers of child contacts younger than five years who were reported, screened for active disease, found eligible for preventive treatment and started on preventive treatment (Fig. 2). The data subsequently collected on the revised version of this form allowed the measurement of three WHO-recommended indicators of latent tuberculosis infection: (i) the screening coverage, i.e. the percentage of children reported as close or household contacts of bacteriologically confirmed cases of pulmonary tuberculosis who were checked for active disease; (ii) the treatment initiation proportion, i.e. the percentage of children found negative for active disease and considered eligible for preventive treatment who had actually initiated such treatment; and (iii) the proportion of children that completed preventive treatment.^7^

### Regional pilot study

In a pilot study in the Hauts-Bassins region of Burkina Faso, we investigated how the replacement of the routine passive system of contact tracing with active household-based tracing by community health workers (CHWs) would affect the numbers of children deemed eligible for preventive treatment. Hauts-Bassins was purposefully selected as the study area for its convenience. In 2016, it was the region of Burkina Faso with the second highest number of contagious tuberculosis cases, after the Central region (A Combary, National Tuberculosis Programme of Burkina Faso, unpublished data, 2016). The estimated population of Hauts-Bassins in 2016 was 2 025 513 or about 10.6% of the total population of Burkina Faso.^11^ The region includes both rural and urban districts and probably holds about 10% of the country’s CHWs, who collaborate actively with local civil society organizations.

We compared data collected on tuberculosis during August and September of 2016, when active tracing of contacts by CHWs was implemented, with the corresponding data collected, in the same area, in June and July of 2016, when all contact tracing relied on the national system of passive detection. For the active tracing, CHWs were trained to visit the households of all bacteriologically confirmed cases of pulmonary tuberculosis, identify all children within the household who were younger than five years and educate the parents or caregivers of such children on the importance of screening child contacts for tuberculosis. Eventually, a tuberculosis nurse visited the household of each known confirmed case, checked all exposed children for the signs or symptoms of tuberculosis, referred all suspected paediatric cases of active tuberculosis to the nearest centre for diagnosis and treatment and started to give preventive treatment, directly within the household, to all eligible children. Our outcomes of interest were screening coverages, proportion of children starting on treatment and the numbers of contacts younger than five years identified per confirmed index case, known as the contact:index ratios.

We used the contact:index ratio estimated during the pilot study’s active contact tracing to assess the effect that the national reliance on passive contact tracing had on the cascade of care for latent tuberculosis infection, and the corresponding WHO-recommended indicators.

### Data analysis

Descriptive statistics were used to summarize observations made, during our main study period and with the revised latent tuberculosis infection register and quarterly tuberculosis reporting form, in each of Burkina Faso’s 13 regions and nationwide. Since the pilot study was not intended to be a detailed statistical comparison, the analysis of the effect of active contact tracing on common indicators of latent tuberculosis infection was also descriptive.

## Results

### Monitoring and evaluation system

During the study period, the revised registration form was rapidly adopted in every centre for tuberculosis diagnosis and treatment and all scheduled revised quarterly reporting forms were completed by each such centre. Over the same period, 3717 cases of pulmonary tuberculosis were bacteriologically confirmed in Burkina Faso and 1166 contacts younger than five years were reported. The corresponding contact:index ratio was therefore 0.31 nationwide. However, the regional values for this ratio varied from 0.04 in the Sahel region to 0.46 in the Boucle de Mouhoun region.

Of the 1166 contacts younger than five years that were reported during the study period, 956 (82.0%) were checked for active tuberculosis. Regionally, screening coverage varied from 57.1% (164/287) in Hauts-Bassins to 100% (15/15) in the Sahel region.

Of the 956 children who were reportedly checked for active tuberculosis, 941 (98.4%) were declared eligible for isoniazid preventive therapy. The reasons why 15 children were declared ineligible were not reported. During the study period, 64 children younger than five years old were reported as new cases of active tuberculosis.

As 852 children were reported to have begun preventive treatment during our main study period, the overall proportion of children initiating treatment was 90.5% (852/941). Regionally, such proportions varied from 57.1% (20/35) in Cascases region to 100% (149/149) in the Nord region. According to information collected during routine supervisory visits, the main reason for not starting treatment was a temporary interruption in the isoniazid supply at health-facility level.

### Pilot study

**Fig. 3:**
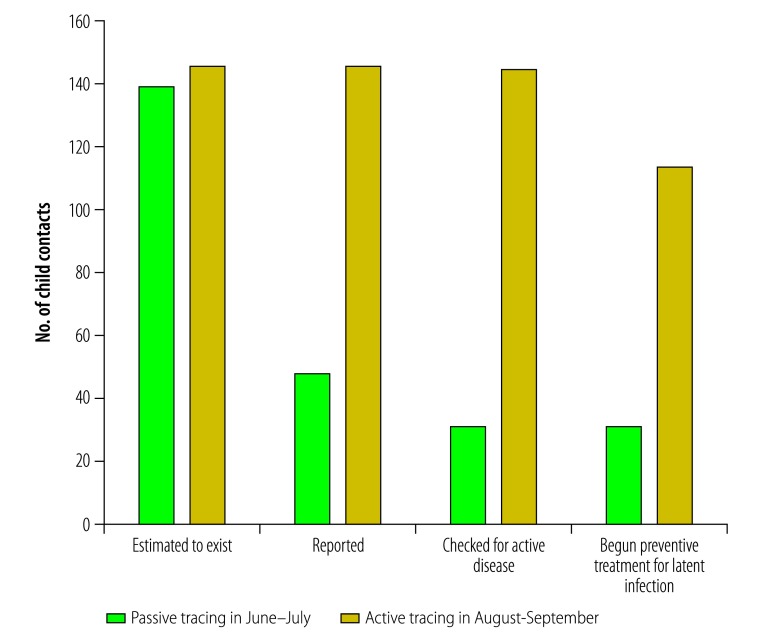
Child contacts of active cases of tuberculosis: estimated numbers and numbers reported, checked for active disease and begun on preventive treatment, Hauts-Bassins, Burkina Faso, 2016

Notes: The contacts investigated were younger than five years and identified by routine passive tracing in June–July 2016 and by active tracing, by community health workers who visited the households of active cases, in August–September 2016.

Although the type of contact tracing appears to have had little, if any, effect on the numbers of active index cases reported, i.e. 76 with passive tracing and 80 with active tracing, the use of active tracing appears to have markedly increased the reported number of child contacts younger than five years, i.e. from 48 with passive tracing to 146 with active tracing (Fig. 3). The contact:index ratio for the active-tracing phase of the study was therefore much higher than that for the passive-tracing phase: 1.83 vs 0.63. The percentage of index cases found to have at least one exposed child younger than five years in their household also increased, from 23.7% (18/76) in the passive-tracing phase to 63.8% (51/80) in the active-tracing phase. However, the corresponding mean number of children younger than five years reported in a household with an index case remained more-or-less unchanged: 2.67 vs 2.86.

Although screening coverage appeared markedly lower in the passive-tracing phase than in the active-tracing phase, 64.6% (31/48) vs 99.3% (145/146), the proportion of eligible children initiating treatment showed the opposite trend – 100% (31/31) vs 78.6% (114/145). Again, supervisory visits revealed that most failures to start preventive treatment were the result of the unavailability of isoniazid.

If we assume that the 1.83 contact:index ratio recorded in the active-tracing phase of the pilot study was a true estimate of the contact:index ratio in the passive-tracing phase, and that all index cases were detected in the earlier phase, then there were 139 contacts younger than five years in Hauts-Bassins during the passive-tracing phase and not the 31 reportedly checked for active disease. The true screening coverage during the passive-tracing phase may therefore have been only about 22.3% (31/139), i.e. only about a quarter of the reported proportion.

## Discussion

By adapting tuberculosis recording and reporting tools to capture data on latent tuberculosis infection, the national tuberculosis programme was able to measure and report screening coverages and proportions of children initiating treatment for contacts younger than five years.

Our main study was limited to data collected routinely, using the revised national reporting system. The revised system proved to be feasible and allowed nationwide measurement of core global and national indicators, for activities related to latent tuberculosis infection, under routine programmatic conditions. No additional personnel were required and training sessions specifically focused on activities related to latent tuberculosis infection, including recording, reporting, monitoring and evaluation, were easily included in the general training programmes organized by the national tuberculosis programme.

National values for screening coverage (82.0%) and the proportion of treatment initiation (90.5%) recorded over our main study period indicated that, at least in Burkina Faso and provided that political commitment is maintained, the targets of the End TB Strategy are within reach. Although we do not report any treatment completion proportions, historical data from Burkina Faso indicate that about 81% of children who start isoniazid preventive treatment also complete it (A Combary, National Tuberculosis Programme of Burkina Faso, unpublished data, 2016). Among the children of Burkina Faso, therefore, failure to complete preventive treatment is probably not a major challenge in the cascade of preventive care. Hopefully, uptake of the latest recommendations from WHO on preventive treatment against latent tuberculosis infection in children, i.e. the replacement of six months of isoniazid with three months of rifampicin and isoniazid,^12^ will increase the proportion of children completing treatment.

The global proportion of people initiating treatment estimated by WHO in 2016, 13%, is far from the target of 90% set to be reached by 2025.^6^^,^^7^ The implementation and monitoring of preventive therapy in child contacts need to be scaled up at a rapidly increasing pace. This may be greatly facilitated by the development of better recording and reporting systems like the one described here. The denominators for the global indicators of latent tuberculosis infection are currently predominately represented by crude estimates that can only be improved by the collection of national data of good quality.^7^ Here we provide a model for measuring contact screening coverage, an indicator that many countries might find challenging to capture.

Our pilot research project, on the effect on indicators relating to latent tuberculosis infection of active contact tracing within the households of index cases, revealed that many exposed children were probably being missed by passive contact tracing. Compared with routine passive tracing, the active tracing appears to have resulted in a threefold increase in the absolute number of children initiating preventive treatment. Passive contact tracing, which is currently adopted by most countries in sub-Saharan Africa, may leave as many as two thirds of eligible children without access to screening and treatment.

Unexpectedly, we recorded a lower proportion in treatment initiation in the active-tracing phase than during the passive-tracing phase. This is probably the result of the sudden increase in the number of children considered eligible for treatment, as the result of active tracing, without a simultaneous corresponding increase in the provision of isoniazid. Control programmes attempting to improve contact identification should expect and prepare for an increased demand for preventive treatment. In the centres for diagnosis and treatment that always had isoniazad available, every eligible child that was identified via active tracing started preventive therapy. The active-tracing model that we investigated appears to have been accepted well by both the general population and the health-care workers.

We were also surprised that, in our main study, only 15 out of the 956 children screened were considered ineligible for isoniazid preventive therapy. We expected more exclusions because of active tuberculosis in the screened contacts. However, paediatric tuberculosis can be hard to diagnose, particularly in the early phases of the disease.^4^ New tools for the early detection of tuberculosis in children are clearly required. In this respect, the identification of clinically-suitable tuberculosis biomarkers and the description of transcriptional signatures of incipient disease hold promise, especially for young contacts.^13^^,^^14^

Problems in measuring the first step of the cascade of care for the prevention of tuberculosis, i.e. capturing data on children eligible for screening, can distort the whole cascade. Our data indicate that estimates of screening coverage based on routinely reported data and passive contact tracing may be much higher than the true coverage.

Both our main study and pilot study had limitations. Our feasibility analysis of the monitoring and evaluation system was incomplete as we did not collect data on costs and could not evaluate the system’s cost–effectiveness analysis. The value of our estimate of the benefits of active contact tracing at household level is limited by the short period of implementation, potential seasonal bias in the data and the relatively small sample.

In conclusion, by revising an existing register and reporting form, we facilitated the routine collection of data on activities related to latent tuberculosis infection in a resource-poor country in sub-Saharan Africa. We anticipate that the recording and reporting tools that we developed could be incorporated into current health information systems. Our main study probably had an important side-benefit: the increased sensitization of health managers and health-care workers with regard to the prevention and care of childhood tuberculosis. Our pilot study indicated that many children who were eligible for preventive treatment are being missed as the result of the passive identification and recruitment of exposed children. Research is needed on the feasibility and value of the large-scale implementation of active tracing of contacts younger than five years, by CHWs making household visits and of the effective checking of children for active tuberculosis at peripheral health centres.
